# Identifying Individuals at Risk of Functional Decline Despite Preserved Muscle Mass: Insights from Appendicular Skeletal Muscle Mass and Handgrip Strength Discordance

**DOI:** 10.3390/jcm15072650

**Published:** 2026-03-31

**Authors:** Van-Tuy Nguyen, Yangsik Jeong, Taesic Lee

**Affiliations:** 1 Department of Biochemistry, Yonsei University Wonju College of Medicine, Wonju 26426, Republic of Korea; nvtuy@yonsei.ac.kr; 2 Department of Global Medical Science, Yonsei University Wonju College of Medicine, Wonju 26426, Republic of Korea; 3 Organelle Medicine Research Center, Yonsei University Wonju College of Medicine, Wonju 26426, Republic of Korea; 4 ONCOin, Ltd., Startup Cube #2-204, 1 Kangwondaehak-gil, Chuncheon 24341, Republic of Korea; 5 Institute of Mitochondrial Medicine, Yonsei University Wonju College of Medicine, Wonju 26426, Republic of Korea; 6 Division of Data Mining and Computational Biology, Department of Convergence Medicine, Yonsei University Wonju College of Medicine, Wonju 26426, Republic of Korea

**Keywords:** muscle quality, handgrip strength, skeletal muscle mass, adiposity, aging, sarcopenia

## Abstract

**Background:** Muscle strength is a more robust indicator of functional health than muscle mass alone, yet muscle mass-based assessments remain common. Discordance between appendicular skeletal muscle mass (ASM) and handgrip strength (HGS) reflects impaired muscle quality and may identify individuals at risk of functional decline despite preserved muscle mass. **Methods:** We conducted a cross-sectional analysis of adults aged >18 years from the KNHANES 2022–2023 (N = 4130). Mean ages were 57.7 ± 17.2 years in men and 58.4 ± 16.3 years in women. ASM was measured by bioelectrical impedance analysis and HGS using a standardized dynamometer. ASM–HGS discordance was defined as the residual difference between observed and predicted HGS. Sex-stratified, survey-weighted linear regression models were used to identify associated factors, with dose–response relationships examined across quintiles of age and body fat percentage. **Results:** ASM and HGS were moderately correlated in both men (PCC = 0.615) and women (PCC = 0.567), with substantial interindividual discordance. Greater ASM-HGS discordance was independently associated with older age and higher body fat percentage in both sexes, demonstrating clear dose–response relationships, with progressively greater muscle mass-dominant patterns at higher levels of age and adiposity. Anemia was independently associated with greater discordance in both men and women, with a significant interaction by sex. **Conclusions:** Although ASM and HGS are strongly correlated in Korean adults, clinically meaningful discordance exists in a substantial subset, revealing heterogeneity in muscle quality not captured by muscle mass alone. Excess adiposity and aging are major determinants of this discordance.

## 1. Introduction

Skeletal muscle health is a central determinant of physical function, independence, and long-term survival across the adult lifespan. Although skeletal muscle mass has historically been emphasized as a key marker of musculoskeletal health, a growing body of evidence indicates that muscle strength is more strongly associated with disability, hospitalization, and mortality than muscle quantity alone [1,2]. In other words, while muscle mass and strength share significant physiological and clinical features, they frequently exhibit independent patterns, leading to the concept of dynapenia, defined as the age-related loss of muscle strength that is not fully explained by declines in muscle mass [3,4]. In response, contemporary sarcopenia consensus statements now prioritize muscle strength as the principal diagnostic component, with appendicular skeletal muscle mass (ASM) serving a supportive role [5,6,7]. Nevertheless, muscle mass-centered assessments remain widely used in both clinical practice and population-based research, potentially obscuring functional vulnerability among individuals with preserved muscle mass.

At the population level, ASM and handgrip strength (HGS) demonstrate moderate to strong correlations; however, substantial interindividual variability exists around this relationship [8]. A subset of individuals exhibits disproportionately low strength relative to their muscle mass, reflecting impaired muscle quality-the ability of muscle tissue to generate force per unit mass [9,10]. This pattern is closely related to the concept of dynapenia and highlights that preserved muscle mass does not necessarily translate into preserved muscle function. Such muscle mass–strength discordance represents a clinically meaningful phenotype that is not captured by conventional sarcopenia definitions and may identify individuals at heightened risk of functional decline despite apparently adequate muscle reserves [11].

Excess adiposity has emerged as a major contributor to impaired muscle quality. Epidemiologic studies consistently demonstrate that individuals with higher body fat may have preserved or even greater absolute ASM yet exhibit lower strength relative to muscle mass, indicating reduced specific force rather than true functional hypertrophy [12]. Mechanistically, adiposity is associated with intramuscular adipose infiltration, altered muscle fiber architecture, mitochondrial dysfunction, insulin resistance, and chronic low-grade inflammation, all of which impair contractile efficiency without necessarily reducing muscle mass [13,14]. These deleterious effects appear to be magnified with advancing age, suggesting a synergistic interaction between aging biology and adiposity in driving muscle mass–strength mismatch [15].

Several approaches have been proposed to quantify muscle quality by integrating measures of muscle strength and muscle mass, including strength-to-mass ratios and muscle quality indices [9,16]. However, no consensus metric currently exists for evaluating the relationship between muscle quantity and functional capacity at the population level [17,18]. In epidemiologic studies, ASM—defined as the sum of muscle mass across all four limbs—is widely used as a proxy for total skeletal muscle mass because it represents the major locomotor muscle compartments and correlates strongly with whole-body muscle mass [19,20]. When combined with HGS, which is a validated indicator of overall muscle strength and functional status, this framework provides a practical method for examining discrepancies between muscle quantity and functional performance in large population samples [21,22].

Using nationally representative data from the Korea National Health and Nutrition Examination Survey (KNHANES) 2022–2023 [23], the present study applies an index of ASM-HGS discordance as a practical, population-level indicator of muscle quality. We aimed to characterize population-level patterns of muscle mass–strength discordance, identify its principal determinants, and examine how factors influencing muscle quality vary by sex. By shifting the focus from muscle quantity to functional efficiency, this study seeks to improve identification of individuals at risk of functional decline despite preserved muscle mass.

## 2. Materials and Methods

### 2.1. Study Design and Population

This cross-sectional study analyzed data from the KNHANES 2022–2023, a nationally representative survey conducted using a multistage, stratified probability sampling design. Adults aged >18 years with complete data on ASM, HGS, and key covariates were included. All participants provided informed consent, and KNHANES protocols were approved by the institutional review board of the Korea Disease Control and Prevention Agency [23].

### 2.2. Sample Size

As a secondary analysis of KNHANES, which has a predetermined sample size, a formal priori sample size calculation was not performed. The final analytic sample (N = 4130) was sufficient to support multivariable survey-weighted regression models including 11 covariates, as well as sex-specific and age-stratified analyses.

### 2.3. Assessment of Muscle Mass and Muscle Strength

ASM was calculated as the sum of lean soft tissue mass from the four limbs and expressed in kilograms (kg). Body composition was measured using bioelectrical impedance analysis (BIA) according to the standardized protocol of the KNHANES [23]. BIA has been widely used in large epidemiological studies and provides reliable estimates of appendicular skeletal muscle mass in population-based settings [24,25].

HGS was measured using a digital hand dynamometer (T.K.K. 5401; Takei Scientific Instruments, Tokyo, Japan), which measures force between 5.0 and 100.0 kg in 0.1 kg increments and includes an adjustable grip span. Measurements were performed according to the standardized KNHANES protocol [23]. Participants performed the test in a standing position with the arm fully extended at the side without touching the body. Grip strength was measured twice for each hand alternately, and the mean value of all measurements was used to represent overall HGS. HGS measured using this protocol has been widely validated as a reliable indicator of overall muscle strength in epidemiologic research [26,27].

### 2.4. Covariates and Conceptual Framework

**Figure 1:**
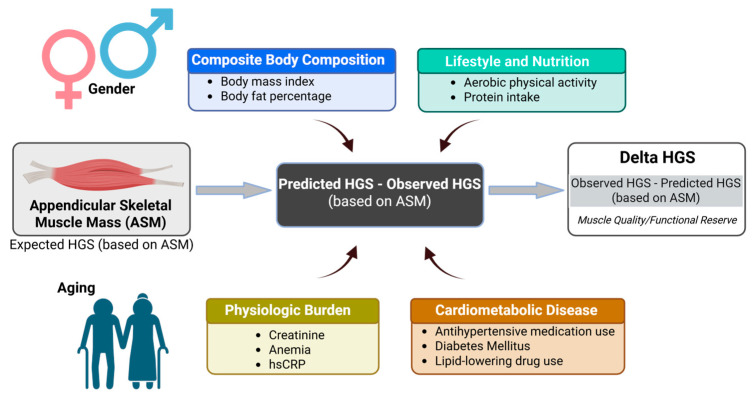
Conceptual framework of factors contributing to ASM-HGS discordance.

Covariates were selected based on prior evidence and biological plausibility, as summarized in Figure 1. These included body composition parameters (body mass index (BMI), body fat percentage), diabetes mellitus (DM), hematologic status (anemia), inflammatory markers (high-sensitivity C-reactive protein (hsCRP)), renal function (serum creatinine), medication use (antihypertension medication (AHM), lipid-lowering drug (LLD)), and protein intake adequacy. Lifestyle factors included aerobic physical activity, muscle-strengthening activity, and combined physical activity, defined as meeting both aerobic and muscle-strengthening recommendations [28]. hsCRP and serum creatinine were analyzed as categorical variables (elevated vs. non-elevated) based on predefined clinical cutoffs (Table S1).

### 2.5. Statistical Analysis

Continuous variables are presented as unweighted means with standard deviations, and categorical variables as frequencies and percentages. To quantify discordance between ASM and HGS, sex-specific linear regression models were constructed with HGS as the dependent variable and ASM as the predictor. Predicted HGS values were derived from these models, and the residual difference between predicted and observed HGS was calculated for each participant. This residual (∆HGS) represents the degree of discordance between muscle mass and strength. The residual values were used directly without standardization. Participants were then categorized into quintiles of the residual distribution to define five ASM–HGS discordance groups, ranging from relatively HGS-dominant to ASM-dominant phenotypes. Differences across ASM-HGS discordance groups were assessed using linear regression for continuous variables and logistic regression for categorical variables, with discordance groups modeled as an ordinal variable to evaluate linear trends.

Associations between clinical factors and ASM-HGS discordance were examined using sex-specific linear regression models. Univariable analyses were first conducted to explore crude associations. Multivariable models were then constructed, including variables with significant univariable associations together with covariates selected a priori based on prior literature and biological plausibility (Figure 1). In addition, clinically relevant variables were also evaluated within the multivariable framework even if they were not statistically significant in univariable analysis to ensure appropriate control for potential confounding.

Dose–response relationships were further evaluated by dividing age and body fat percentage into survey-weighted quintiles. Quintiles were used to assess potential linear trends across the population distribution because clinically established thresholds for these variables in relation to ASM–HGS discordance have not been defined [29]. Compared with tertiles, quintiles provide greater resolution for evaluating potential dose–response relationships while maintaining adequate sample size within each category. In addition, the use of an odd number of categories allows the identification of a conceptually balanced middle group. Multivariable survey-weighted linear regression models were then fitted to estimate adjusted regression coefficients across quintile categories, and linear trends were assessed by modeling the median value of each quintile as a continuous variable. Effect modification by sex was assessed by including interaction terms in multivariable models.

All regression analyses accounted for the complex, multistage sampling design of the survey by incorporating sampling weights, stratification, and clustering. Statistical analyses were conducted using R software version 4.4.2. All tests were two-sided, and a *p* value < 0.05 was considered statistically significant.

## 3. Results

### 3.1. Participant Characteristics Across ASM-HGS Discordance Groups

To identify factors related to the discordance between ASM and HGS in Korean adults, we analyzed data from the 2022–2023 KNHANES, which included 1934 men and 2196 women. Although the study included all adults aged >18 years according to the survey design, the age distribution of the cohort was skewed toward middle-aged and older individuals. The mean ages were 57.7 ± 17.2 years for men and 58.4 ± 16.3 years for women (Figure S1). HGS exhibited a Gaussian distribution in both sexes, with mean values of 35.9 ± 7.64 kg in men and 21.8 ± 4.64 kg in women (Figure S2). Similarly, ASM demonstrated normality, with mean values of 22.4 ± 3.62 kg in men and 15.2 ± 2.4 kg in women (Figure S3). Given the well-established sex-specific differences in muscle mass and strength, and the potential for sex to act as an effect modifier in the association between ASM and HGS, all subsequent analyses were conducted separately for men and women. Stratified multivariable regression models were constructed using identical covariate adjustment strategies within each sex.

**Figure 2:**
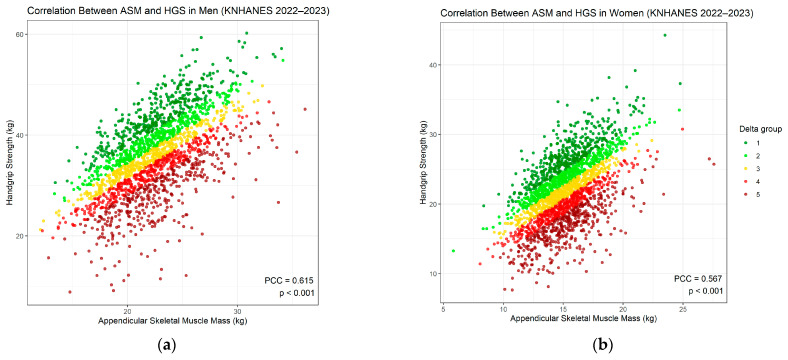
Correlation between ASM and HGS in men and women: (**a**) Pearson correlation between ASM and HGS in men. (**b**) Pearson correlation between ASM and HGS in women.

In the preliminary analysis, Pearson correlation between ASM and HGS revealed significant positive relationships in both men and women (PCC = 0.615 in men and 0.567 in women; Figure 2). In both sexes, linear regression of HGS on ASM demonstrated an elliptical distribution of data points surrounding the regression line. A similar elliptical clustering pattern between muscle mass and strength has been reported in previous epidemiologic studies, reflecting the general proportional relationship between these two parameters [10,30,31]. However, a considerable number of observations were located outside the expected elliptical boundary, indicating that mass–strength discordance was more common than anticipated. To quantify this gap between muscle mass and strength, we calculated the delta between predicted and observed HGS (by sex-specific linear regression and ASM), which may serve as an index of muscle quality. This index quantifies how closely the observed muscle strength aligns with the level predicted from muscle mass, such that values near the regression line indicate high concordance, whereas greater deviations from the line indicate greater discordance. Two types of discordance cases could occur; the first corresponds to the red-to-brown shaded region (Figure 2), indicating an HGS-recessive group, which included individuals with weaker HGS than would be expected for their muscle mass. The other discordant pattern corresponds to the green-shaded region, representing an HGS-dominant group, which is presumed to be metabolically favorable (Figure 2).

Table 1 summarizes general characteristics according to five groups clustered by the degree of correlation between HGS and muscle mass (Table 1). In the Korean men, the following clinical and metabolic linear trends across groups from Group 1 (HGS-dominant) to Group 5 (HGS-recessive) were exhibited: older age, higher BMI, greater body fat percentage, lower levels of aerobic physical activity, higher prevalence of AHM use, DM, LLD use, higher prevalence of anemia, increase creatinine, as well as less protein intake. While women exhibited similar monotonic trends for all clinical variables significant in men, hsCRP showed a significant linear trend across increasing discordance groups only in women (*p* for trend < 0.001) (Table 1). In addition, muscle-strengthening activity and combined physical activity (aerobic + muscle-strengthening) showed significant decreasing trends across increasing discordance groups in men (both *p* for trend < 0.05) (Table S2).

### 3.2. Sex-Specific Factors Associated with ASM-HGS Discordance

Sex-stratified linear regression analyses were conducted to identify factors associated with ASM-HGS discordance (Table 2). All subsequent analyses were performed using the KNHANES complex sampling design, derived from sampling weights, stratification, and clustering. In Korean men, univariable analyses showed that older age, higher BMI, greater body fat percentage, the presence of cardiometabolic comorbidities—defined by the use of AHM or LLD and by diagnoses of DM, anemia, or impaired renal function based on serum creatinine—lower levels of aerobic physical activity, and lower protein intake were all significantly associated with an ASM-dominant pattern of discordance. Moreover, both muscle-strengthening activity and combined physical activity (aerobic + muscle-strengthening) were inversely associated with ASM–HGS discordance, with all regression coefficients showing negative directions and statistical significance in univariable analyses (Table S3). The survey-weighted prevalence of aerobic physical activity and anemia across ASM-HGS discordance groups is presented in Figures S4 and S5, respectively. In women, the same factors were similarly associated with higher ASM–HGS discordance index values. In addition, elevated hsCRP concentrations were significantly associated with higher ASM–HGS discordance index values only among women, suggesting that the previously reported finding that chronic micro-inflammation is a major factor in deteriorating muscle quality is particularly evident in women [32,33].

In weighted survey–based multivariable analyses, older age, higher body fat percentage, and anemia remained independently associated with ASM-dominant in Korean men. In women, comparable independent associations were observed, with lower physical activity levels additionally linked to a shift toward muscle mass–dominant discordance (Table 2).

### 3.3. Linear Trends of Determinants Across ASM-HGS Discordance Groups

Weighted univariable and multivariable linear regression models showed that older age and higher body fat percentage were independently associated with higher ASM-HGS discordance in both sexes. To further explore dose–response relationships, age and body fat percentage were categorized into five quintiles and examined using multivariable-adjusted weighted regression models, with ASM-HGS discordance modeled as an ordinal outcome ranging from group 1 (HGS-dominant) to group 5 (ASM-dominant), where higher values indicate greater relative dominance of ASM over HGS.

**Figure 3:**
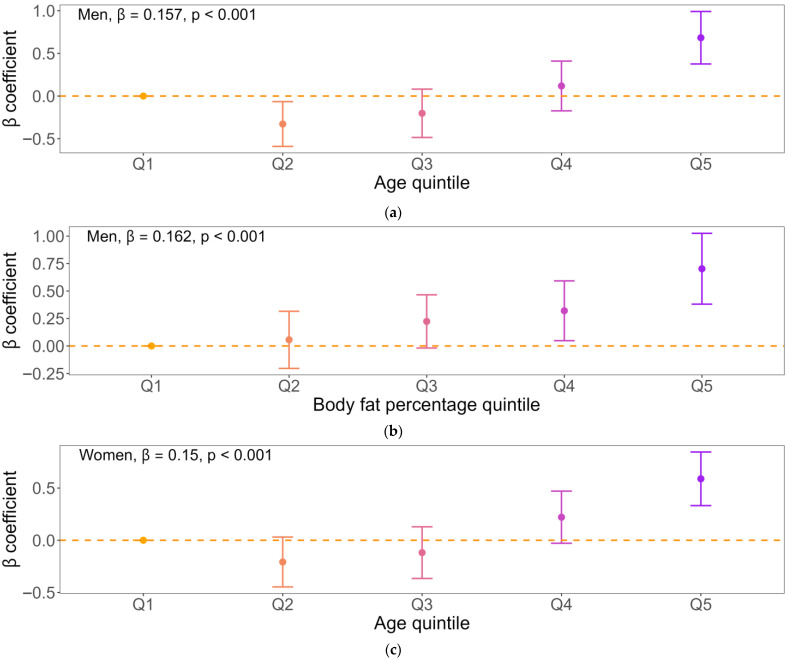

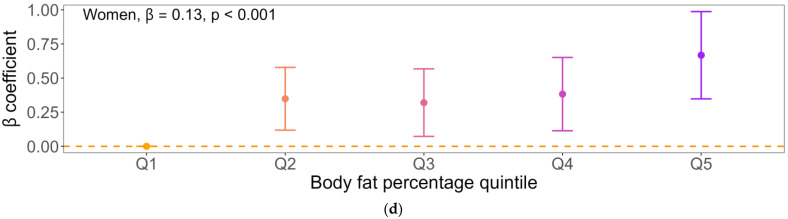
Multivariable-adjusted associations of age, body fat percentage, hemoglobin, and total cholesterol with ASM-HGS discordance. Panels show the associations between (**a**) age and ASM–HGS discordance in men, (**b**) body fat percentage and ASM–HGS discordance in men, (**c**) age and ASM–HGS discordance in women, (**d**) body fat percentage and ASM–HGS discordance in women, β-coefficients (points) and 95% confidence intervals (error bars) are presented for each quintile (Q1–Q5) of the exposure variables, estimated using multivariable linear regression models adjusted for all covariates that were statistically significant in univariable analyses. Point colors indicate quintile order using a continuous gradient, ranging from orange (Q1, lowest) to purple (Q5, highest), with intermediate colors representing Q2–Q4.

Among men, age showed a significant positive linear association with increasing ASM-HGS discordance across quintiles (Figure 3a). Regression coefficients increased progressively from the lowest to the highest age quintile, and trend analysis confirmed a robust dose–response relationship (β for trend = 0.157, *p* < 0.001), indicating a shift toward ASM-dominant discordance with advancing age. Similarly, body fat percentage demonstrated a significant positive linear association with ASM-HGS discordance across quintiles (Figure 3b), with progressively increasing coefficients and a significant trend (β for trend = 0.162, *p* < 0.001), suggesting that greater adiposity is consistently associated with ASM-dominant discordance in men.

Among women, age likewise exhibited a significant positive linear relationship with ASM-HGS discordance across quintiles (Figure 3c), with a clear dose–response pattern (β for trend = 0.150, *p* < 0.001). Body fat percentage also showed a significant positive linear association (Figure 3d), with steadily increasing coefficients across quintiles and a significant trend (β for trend = 0.130, *p* < 0.001), indicating that higher adiposity is associated with greater ASM-dominant discordance in women. To assess the robustness of these findings, sensitivity analyses were conducted using alternative categorizations of age and body fat percentage. When variables were categorized into septiles and noviles, the associations between age, body fat percentage, and ASM–HGS discordance remained consistent, demonstrating similar dose–response patterns in both sexes (Figures S6 and S7).

### 3.4. Interaction Effects of Sex on Determinants of ASM-HGS Discordance

Interaction analyses were conducted to evaluate whether the associations between selected determinants and ASM-HGS discordance differed by sex (Table 3). Among the variables examined, including age, body fat percentage, aerobic physical activity, and LLD use, no statistically significant interactions with sex were observed (all *p* for interaction > 0.05), indicating broadly comparable associations in men and women for these factors.

In contrast, a significant effect modification by sex was identified for anemia (*p* for interaction < 0.05). The interaction term between sex and anemia was negative (β = −0.361), indicating that the association between anemia and ASM-HGS discordance differed in direction by sex. Specifically, anemia was positively associated with higher ASM-HGS discordance in men, whereas this association was attenuated in women. These findings suggest sex-specific biological mechanisms linking hemoglobin status to muscle mass–strength balance, underscoring the importance of considering sex-stratified effects when evaluating hematologic determinants of muscle quality.

## 4. Discussion

In this nationally representative study of Korean adults, we found that, although ASM and HGS exhibited a moderate to strong overall correlation at the population level, consistent with prior reports in Korean and Asian cohorts, a substantial proportion of individuals displayed meaningful discordance between these measures. This discordance reflects clinically relevant heterogeneity in muscle quality (i.e., strength relative to mass) that is not adequately captured by conventional muscle mass-based assessments alone. Specifically, while preserved muscle quantity does not invariably correspond to preserved functional capacity, a notable subset of participants showed disproportionate muscle mass relative to strength (or vice versa), highlighting the limitations of relying solely on ASM for evaluating neuromuscular health and sarcopenia risk. These observations align with emerging evidence that muscle quality, beyond mere quantity, plays a critical role in musculoskeletal outcomes and underscore the need for integrated assessments incorporating both mass and strength metrics in population-based research and clinical practice [30,34,35].

In survey-weighted multivariable analyses, body fat percentage demonstrated a significant positive linear relationship with ASM-HGS discordance across quintiles, with progressively greater ASM-dominant discordance observed at higher levels of adiposity. These findings indicate that increasing adiposity is consistently associated with a disproportionate increase in muscle mass relative to strength, reflecting reduced muscle quality rather than a simple quantitative deficit in lean mass. This interpretation aligns with prior evidence showing that individuals with higher adiposity may preserve or even exhibit greater absolute lean mass while demonstrating reduced muscle quality and lower specific strength [12,36]. Proposed mechanisms include intramuscular fat infiltration, impaired mitochondrial function, altered neuromuscular efficiency, and chronic low-grade inflammation [13,14]. Our results extend this literature by demonstrating that adiposity-related impairments in muscle quality follow a graded, population-level pattern when assessed using an ASM–HGS discordance framework.

Age was also positively and linearly associated with increasing ASM-HGS discordance across quintiles in both sexes. This finding is consistent with the concept of dynapenia, in which age-related declines in neuromuscular function, motor unit remodeling, and excitation-contraction coupling lead to disproportionate losses in muscle strength relative to muscle mass [4,35,37]. Importantly, the persistence of this association after multivariable adjustment suggests that aging contributes to impaired muscle quality beyond its effects on body composition alone.

A significant interaction between anemia and sex emerged in our analysis, revealing that men demonstrated a more pronounced association between anemia and ASM–HGS discordance compared to women. Anemia fundamentally compromises skeletal muscle function through diminished oxygen delivery and impaired oxidative metabolism, culminating in skeletal muscle hypoxia, disrupted mitochondrial activity, and reduced contractile efficiency—effects that disproportionately affect muscle strength relative to mass [38,39]. The heightened vulnerability observed in men likely stems from the shared dependence of both erythropoiesis and skeletal muscle homeostasis on sex hormones, particularly testosterone, which simultaneously drives red blood cell production and sustains muscle strength and quality [40]. In aging men, anemia frequently coincides with progressive androgen decline, and this synergistic deterioration accelerates functional muscle loss while amplifying ASM–HGS discordance [41]. Women, by contrast, appear to possess inherent biological resilience against anemia-related muscle dysfunction, partly attributable to estrogen’s protective role in preserving mitochondrial integrity and attenuating oxidative stress during hypoxic states [42]. Additionally, the physiological adaptations women develop in response to lower baseline hemoglobin concentrations throughout their reproductive years may confer lasting metabolic advantages, rendering their skeletal muscle less susceptible to functional decline when confronted with mild-to-moderate anemia in later life [43].

Lifestyle-related factors, particularly combined physical activity (aerobic + muscle-strengthening), along with muscle-strengthening activity alone and adequate protein intake, were associated with a more favorable (HGS-dominant) pattern in univariable analyses. These findings are consistent with current recommendations from the World Health Organization, which emphasize the combined role of aerobic and resistance exercise in maintaining muscle function and quality [28]. However, these associations were attenuated after multivariable adjustment. This suggests that their beneficial effects on muscle quality may be mediated, at least in part, through favorable influences on adiposity and metabolic health rather than direct effects on muscle mass–strength coupling alone [44,45].

This integrated approach reflects the broader shift toward functional anthropometric methods in sarcopenia research, combining traditional muscle mass quantification (e.g., via BIA, calf circumference, or mid-upper arm circumference) with functional strength measures such as HGS to better evaluate neuromuscular integrity beyond mass alone [46,47]. Anthropometric indicators such as calf and mid-upper arm circumferences have been proposed as practical proxies for skeletal muscle mass and show moderate correlations with muscle strength in population studies [48]. These approaches may offer useful alternatives for large-scale epidemiological research where imaging-based techniques are not feasible.

Muscle-specific strength, defined as grip strength normalized to arm lean mass, has been shown to predict physical performance decline and adverse metabolic profiles, highlighting discordance not captured by total ASM alone [49]. Other studies demonstrate that upper-limb lean mass correlates with grip strength and functional outcomes, supporting the use of regional muscle measures to assess muscle quality in older adults [50]. However, the primary aim of this study was to evaluate whole-body functional capacity rather than isolated upper-limb muscle quality. ASM, defined as the sum of muscle mass from all four limbs, is widely used as a surrogate of total skeletal muscle mass in epidemiologic research and is recommended in major sarcopenia guidelines (e.g., EWGSOP2 and AWGS) [6,29]. Therefore, total ASM was used to represent overall muscle quantity while HGS served as a practical indicator of global muscle strength. This approach allows the ASM–HGS discordance framework to reflect whole-body muscle function rather than regional muscle performance and is consistent with evidence that HGS correlates strongly with overall strength and predicts adverse outcomes across populations [51].

Several strengths of this study warrant emphasis, including the use of a large, nationally representative sample, standardized measurements, and a residual-based approach to quantifying muscle mass–strength discordance that is intuitive and adaptable to other populations. Nevertheless, several limitations should be acknowledged. First, the cross-sectional design precludes causal inference, and reverse causality cannot be excluded. Second, ASM was assessed using BIA rather than imaging-based modalities such as dual-energy X-ray absorptiometry (DXA) or computed tomography, which are considered more precise for evaluating body composition. Therefore, some degree of measurement error in the estimation of muscle mass cannot be excluded and may have influenced the assessment of ASM–HGS discordance. However, BIA is widely used in large population-based surveys, including KNHANES, because of its feasibility and practicality [52,53]. In addition, the EWGSOP2 guidelines recognize BIA as a valid and practical method for estimating muscle mass in clinical and epidemiological settings when imaging-based techniques are not available [7]. Finally, direct measures of muscle composition, neuromuscular function, and longitudinal outcomes were not available in this dataset.

## 5. Conclusions

In this nationally representative sample of Korean adults, although ASM and HGS demonstrated a strong overall correlation and clear linear trends, a non-negligible proportion of individuals exhibited meaningful deviations from this relationship. This discordance underscores considerable heterogeneity in muscle quality, defined as strength relative to mass, which is not adequately captured by relying solely on measures of muscle mass. Advancing age and excess adiposity consistently shifted this discordance toward an inefficient muscle phenotype, indicating preserved muscle quantity without proportional strength. These findings suggest that considering the balance between muscle mass and strength may provide additional insight into muscle health. However, because this study is cross-sectional and does not include functional outcomes such as falls, disability, hospitalization, or mortality, the clinical significance of the ASM–HGS discordance index remains uncertain. Longitudinal studies incorporating functional endpoints are needed to determine their potential prognostic and clinical utility.

## Figures and Tables

**Table 1:** Demographic, clinical, and biochemical characteristics of participants according to ASM-HGS discordance groups.

ASM-HGS Discordance Group		1	2	3	4	5	*p*-for Trend
Age (mean (SD)) (year)	Men	51.28 (15.58)	55.99 (16.14)	58.89 (17.18)	61.18 (16.53)	60.99 (18.52)	<0.001
	Women	53.49 (15.41)	57.64 (15.85)	58.87 (15.75)	60.41 (16.58)	61.40 (16.66)	<0.001
BMI (mean (SD)) (kg/m^2^)	Men	24.43 (3.16)	24.67 (3.39)	24.60 (3.63)	24.48 (3.20)	25.59 (3.80)	<0.001
	Women	23.46 (3.30)	23.86 (3.72)	23.92 (3.36)	24.40 (3.59)	25.30 (3.96)	<0.001
Body fat percentage (mean (SD))	Men	23.98 (5.03)	24.99 (5.26)	25.48 (5.45)	25.97 (5.52)	27.31 (5.71)	<0.001
	Women	32.53 (5.41)	34.01 (5.76)	34.30 (5.46)	35.21 (5.59)	36.27 (5.58)	<0.001
Aerobic physical activity (n, %)	Men	198 (51.2)	181 (46.8)	177 (45.9)	187 (48.3)	139 (35.9)	<0.001
	Women	217 (49.3)	185 (42.1)	178 (40.5)	150 (34.2)	154 (35.1)	<0.001
AHM (n, %)	Men	79 (20.4)	119 (30.7)	135 (35.0)	146 (37.7)	176 (45.5)	<0.001
	Women	106 (24.1)	117 (26.7)	137 (31.2)	149 (33.9)	166 (37.8)	<0.001
DM (n, %)	Men	30 (7.8)	63 (16.3)	61 (15.8)	68 (17.6)	85 (22.0)	<0.001
	Women	30 (6.8)	54 (12.3)	45 (10.3)	49 (11.2)	70 (15.9)	<0.001
LLD (n, %)	Men	53 (13.7)	80 (20.7)	83 (21.5)	92 (23.8)	86 (22.2)	0.002
	Women	103 (23.4)	115 (26.2)	134 (30.5)	160 (36.4)	153 (34.9)	<0.001
Anemia (n, %)	Men	14 (3.6)	18 (4.7)	26 (6.7)	38 (9.8)	54 (14.0)	<0.001
	Women	40 (9.1)	48 (10.9)	49 (11.2)	69 (15.7)	68 (15.5)	<0.001
Evaluation hsCRP (n, %)	Men	128 (33.1)	117 (30.2)	140 (36.3)	129 (33.3)	148 (38.2)	0.078
	Women	109 (24.8)	132 (30.1)	129 (29.4)	146 (33.3)	164 (37.4)	<0.001
Evaluation creatinine (n, %)	Men	15 (3.9)	19 (4.9)	24 (6.2)	35 (9.0)	40 (10.3)	<0.001
	Women	7 (1.6)	11 (2.5)	8 (1.8)	16 (3.6)	16 (3.6)	0.032
Protein intake adequacy (n, %)	Men	220 (56.8)	215 (55.6)	192 (49.7)	194 (50.1)	170 (43.9)	<0.001
	Women	199 (45.2)	190 (43.3)	182 (41.5)	168 (38.3)	146 (33.3)	<0.001

Discordance groups are defined by quintiles of residuals from linear regression of HGS on ASM. *p* for trend is assessed using survey-weighted linear or logistic regression (BMI, body mass index; AHM, antihypertensive medication; DM, diabetes mellitus; LLD, lipid-lowering drug; hsCRP, high-sensitivity C-reactive protein).

**Table 2:** Sex-specific univariate and multivariate linear regression analyses of factors associated with ASM-HGS discordance.

	Men				Women			
	Univariate		Multivariate		Univariate		Multivariate	
	β (95%CI)	*p*	β (95%CI)	*p*	β (95%CI)	*p*	β (95%CI)	*p*
Age (year)	0.015 (0.01–0.02)	<0.001	0.01 (0.004–0.017)	0.002	0.014 (0.009–0.019)	<0.001	0.009 (0.004–0.014)	<0.001
BMI (kg/m^2^)	0.029 (0.007–0.051)	0.009	−0.01 (−0.045–0.025)	0.578	0.066 (0.046–0.086)	<0.001	0.001 (−0.034–0.036)	0.962
Body fat (%)	0.049 (0.038–0.06)	<0.001	0.049 (0.031–0.067)	<0.001	0.054 (0.042–0.067)	<0.001	0.044 (0.022–0.066)	<0.001
Aerobic physical activity	−0.236 (−0.381–−0.09)	0.002	−0.079 (−0.227–0.068)	0.293	−0.317 (−0.47–−0.165)	<0.001	−0.153 (−0.301–−0.004)	0.045
AHM	0.464 (0.302–0.626)	<0.001	0.065 (−0.137–0.267)	0.531	0.383 (0.228–0.538)	<0.001	0 (−0.162–0.163)	0.996
DM	0.429 (0.219–0.639)	<0.001	0.141 (−0.074–0.357)	0.199	0.355 (0.136–0.573)	0.002	0.086 (−0.119–0.291)	0.41
LLD	0.298 (0.115–0.481)	0.002	−0.07 (−0.283–0.143)	0.521	0.366 (0.208–0.524)	<0.001	0.088 (−0.082–0.258)	0.314
Anemia	0.792 (0.517–1.067)	<0.001	0.531 (0.245–0.816)	<0.001	0.257 (0.042–0.472)	0.02	0.208 (0.004–0.412)	0.046
Evaluation hsCRP	0.116 (−0.041–0.272)	0.15	-	-	0.269 (0.124–0.414)	<0.001	0.082 (−0.07–0.234)	0.291
Evaluation creatinine	0.388 (0.055–0.72)	0.023	0.064 (−0.251–0.378)	0.692	0.504 (0.114–0.895)	0.012	0.044 (−0.331–0.419)	0.818
Protein intake adequacy	−0.231 (−0.393–−0.07)	0.005	−0.096 (−0.257–0.065)	0.245	−0.244 (−0.378–−0.11)	<0.001	−0.048 (−0.185–0.088)	0.488

Survey-weighted univariate and multivariate linear regression analyses; results are β (95% CI) (BMI, body mass index; AHM, antihypertensive medication; DM, diabetes mellitus; LLD, lipid-lowering drug; hsCRP, high-sensitivity C-reactive protein).

**Table 3:** Interaction effects of sex on the associations between factors and ASM-HGS discordance.

	Interaction β (95%CI)	*p* for Interaction
Age (year)	−0.001 (−0.007–0.004)	0.669
Body fat (%)	0.001 (−0.015–0.018)	0.867
Aerobic physical activity	−0.052 (−0.243–0.139)	0.594
LLD	0.063 (−0.159–0.285)	0.576
Anemia	−0.361 (−0.700–−0.022)	0.038

Survey-weighted multivariate linear regression models with sex interaction terms; interaction β (95% CI) and Wald test *p* values are shown (LLD, lipid-lowering drug).

## Data Availability

The data presented in this study are publicly available from the Korea National Health and Nutrition Examination Survey (KNHANES) 2022–2023 [13], available at https://knhanes.kdca.go.kr (accessed on 20 December 2025).

## Supplementary Materials

The following supporting information can be downloaded at: https://www.mdpi.com/article/10.3390/jcm15072650/s1, Table S1. Definitions and Operationalization of Study Variables. Table S2. Distribution of Muscle-Strengthening, and Combined Physical Activity Across ASM–HGS Discordance Groups. Table S3. Sex-Specific Univariable Linear Regression Analyses of Physical Activity Variables in Relation to ASM–HGS Discordance. Figure S1. Age distribution of the study population by sex. Figure S2. Distribution of handgrip strength by sex. Figure S3. Distribution of appendicular skeletal muscle mass by sex. Figure S4. Survey-weighted prevalence of aerobic physical activity according to ASM–HGS discordance group, stratified by sex. Figure S5. Survey-weighted prevalence of anemia according to ASM–HGS discordance group, stratified by sex. Figure S6. Multivariable-adjusted associations of age, body fat percentage, hemoglobin, and total cholesterol with ASM-HGS discordance (septile). Figure S7. Multivariable-adjusted associations of age, body fat percentage, hemoglobin, and total cholesterol with ASM-HGS discordance (novile).

## Abbreviations

The following abbreviations are used in this manuscript:

ASM	Appendicular Skeletal Muscle Mass
HGS	Handgrip Strength
KNHANES	Korea National Health and Nutrition Examination Survey
BIA	Bioelectrical Impedance Analysis
BMI	Body Mass Index
AHM	Antihypertensive Medication
DM	Diabetes Mellitus
LLD	Lipid-Lowering Drug
hsCRP	High-Sensitivity C-Reactive Protein
PCC	Pearson Correlation Coefficient
