# Supplementary material for: Identifying Individuals at Risk of Functional Decline Despite Preserved Muscle Mass: Insights from Appendicular Skeletal Muscle Mass and Handgrip Strength Discordance
Source: J Clin Med. 2026 Mar 31;15(7):2650. doi: 10.3390/jcm15072650 (PMC13073496; doi:10.3390/jcm15072650)
Supplement: Supplementary file 1 [file jcm-15-02650-s001.zip › jcm-4195684-supplementary.pdf]

Supplementary Materials for

Identifying Individuals at Risk of Functional Decline Despite Preserved Muscle Mass: Insights from Appendicular Skeletal Muscle Mass and Handgrip Strength Discordance

Van-Tuy Nguyen [1,2,3], Yangsik Jeong [1,2,3,4,5,*] and Taesic Lee [3,6,*]

1 Department of Biochemistry, Yonsei University Wonju College of Medicine, Wonju 26426, Republic of Korea; nvtuy@yonsei.ac.kr

2 Department of Global Medical Science, Yonsei University Wonju College of Medicine, Wonju 26426, Republic of Korea

3 Organelle Medicine Research Center, Yonsei University Wonju College of Medicine, Wonju 26426, Republic of Korea

4 ONCOin, Ltd., Startup Cube #2-204, 1 Kangwondaehakgil, Chuncheon 24341, Republic of Korea

5 Institute of Mitochondrial Medicine, Yonsei University Wonju College of Medicine, Wonju 26426, Republic of Korea

6 Division of Data Mining and Computational Biology, Department of Convergence Medicine, Yonsei University Wonju College of Medicine, Wonju 26426, Republic of Korea

* Correspondence: yjeong@yonsei.ac.kr (Y.J.); ddasic123@yonsei.ac.kr (T.L.); Tel.: +82-102-791-0284 (Y.J.); +82-104-581-1625 (T.L.)

Index

Supplementary Tables 3

Supplementary Table S1. Definitions and Operationalization of Study Variables ... 3

Supplementary Table S2. Distribution of Muscle-Strengthening, and Combined Physical Activity Across ASM–HGS Discordance Groups 5

Supplementary Table S3. Sex-Specific Univariable Linear Regression Analyses of Physical Activity Variables in Relation to ASM–HGS Discordance 6

Supplementary Figures 7

Figure S1. Age distribution of the study population by sex 7

Figure S2. Distribution of handgrip strength by sex 8

Figure S3. Distribution of appendicular skeletal muscle mass by sex 9

Figure S4. Survey-weighted prevalence of aerobic physical activity according to ASM–HGS discordance group, stratified by sex 10

Figure S5. Survey-weighted prevalence of anemia according to ASM–HGS discordance group, stratified by sex 11

Figure S6. Multivariable-adjusted associations of age, body fat percentage, hemoglobin, and total cholesterol with ASM-HGS discordance (septile) 12

Figure S7. Multivariable-adjusted associations of age, body fat percentage, hemoglobin, and total cholesterol with ASM-HGS discordance (novile) 13

Supplementary Tables

Supplementary Table S1. Definitions and Operationalization of Study Variables

Variable	Description
**Appendicular Skeletal Muscle Mass (ASM)**	Sum of appendicular lean soft tissue mass of the right arm, left arm, right leg, and left leg measured by bioelectrical impedance analysis; expressed in kilograms (kg).
**Handgrip Strength (HGS)**	Mean handgrip strength calculated from four valid measurements (two per hand) obtained using a digital dynamometer after exclusion of invalid test records; expressed in kilograms (kg).
**ASM–HGS Discordance Index**	Predicted HGS values were estimated using linear regression models with ASM as the independent variable. The residual difference between observed and predicted HGS was calculated for each participant and used as a continuous index of ASM–HGS discordance: ASM–HGS Discordance=$HGS_{observed}-HGS_{predicted}$<br>Participants were additionally categorized into five groups based on the distribution of this discordance index to facilitate descriptive analyses and assessment of linear trends.
**Age**	Chronological age at the time of survey, expressed in years.
**Sex**	Biological sex classified as men or women based on self-report.
**Body mass index (BMI)**	Body mass index calculated as body weight divided by height squared; expressed in kilograms per square meter (kg/m^2).
**Body fat percentage**	Percentage of total body fat measured by bioelectrical impedance analysis; expressed as a percentage (%).
**Aerobic physical activity**	Binary variable indicating whether participants met recommended aerobic physical activity levels based on the KNHANES questionnaire (≥150 min/week of moderate activity, ≥75 min/week of vigorous activity, or an equivalent combination).

**Muscle-strengthening activity**	Binary variable indicating whether participants met recommended muscle-strengthening activity levels based on the KNHANES questionnaire (⩾2 days per week of strength or resistance exercises).
**Combined physical activity (aerobic + muscle-strengthening)**	Categorical variable indicating combined physical activity status based on aerobic and muscle-strengthening activity. Participants were classified into four groups: (1) neither aerobic nor muscle-strengthening activity, (2) aerobic activity only, (3) muscle-strengthening activity only, and (4) both aerobic and muscle-strengthening activity (meeting combined recommendations consistent with the World Health Organization guidelines).
**Diabetes mellitus (DM)**	Self-reported physician-diagnosed diabetes mellitus.
**Antihypertensive medication use (AHM)**	Regular use of antihypertensive medication assessed by self-reported medication frequency. Participants reporting use daily, ≥20 days per month, or ≥15 days per month were classified as users, whereas those reporting use ≤7 days per month, no use, or unknown frequency were classified as non-users.
**Lipid-lowering drug use (LLD)**	Use of lipid-lowering medication determined from self-reported medication data. Participants reporting current use were classified as users, whereas those reporting no use or unknown status were classified as non-users.
**Anemia**	Low hemoglobin concentration defined as <13.0 g/dL in men and <12.0 g/dL in women; categorized as yes or no.
**Elevated hsCRP**	High inflammatory status defined as hsCRP ≥1.0 mg/L; categorized as yes or no.
**Elevated creatinine**	Elevated serum creatinine defined as >1.2 mg/dL in men and >1.0 mg/dL in women; categorized as yes or no.
**Protein intake adequacy**	Dietary protein intake normalized to body weight, defined as adequate when ≥1.0 g/kg/day; categorized as sufficient or insufficient.

Supplementary Table S2. Distribution of Muscle-Strengthening, and Combined Physical Activity Across ASM–HGS Discordance Groups

ASM-HGS Discordance Group			1	2	3	4	5	p-for trend
Muscle-strengthening activity	Men	<2 days/week	243 (62.8)	256 (66.1)	241 (62.4)	265 (68.5)	282 (72.9)	0.003
		≥2 days/week	144 (37.2)	131 (33.9)	145 (37.6)	122 (31.5)	105 (27.1)	
	Women	<2 days/week	351 (79.8)	358 (81.5)	360 (82.0)	377 (85.9)	380 (86.6)	0.002
		≥2 days/week	89 (20.2)	81 (18.5)	79 (18.0)	62 (14.1)	59 (13.4)	
Combined physical activity	Men	Neither	138 (35.7)	158 (40.8)	137 (35.5)	155 (40.1)	196 (50.6)	<0.001
		Aerobic only	105 (27.1)	98 (25.3)	104 (26.9)	110 (28.4)	86 (22.2)	
		Muscle only	51 (13.2)	48 (12.4)	72 (18.7)	45 (11.6)	52 (13.4)	
		Both	93 (24.0)	83 (21.4)	73 (18.9)	77 (19.9)	53 (13.7)	
	Women	Neither	187 (42.5)	225 (51.3)	233 (53.1)	261 (59.5)	259 (59.0)	<0.001
		Aerobic only	164 (37.3)	133 (30.3)	127 (28.9)	116 (26.4)	121 (27.6)	
		Muscle only	36 (8.2)	29 (6.6)	28 (6.4)	28 (6.4)	26 (5.9)	
		Both	53 (12.0)	52 (11.8)	51 (11.6)	34 (7.7)	33 (7.5)	

Supplementary Table S3. Sex-Specific Univariable Linear Regression Analyses of Physical Activity Variables in Relation to ASM–HGS Discordance

		Men		Women	
		**β (95%CI)**	**p**	**β (95%CI)**	**p**
Muscle-strengthening activity (≥2 days/week)	Yes (vs No)	-0.281 (-0.445, -0.117)	0.001	-0.262 (-0.44, -0.084)	0.004
Combined physical activity (vs Neither)	Aerobic only	-0.189 (-0.389, 0.01)	0.064	-0.335 (-0.503, -0.167)	<0.001
	Muscle only	-0.242 (-0.467, -0.017)	0.036	-0.357 (-0.655, -0.059)	0.019
	Both	-0.424 (-0.626, -0.222)	<0.001	-0.411 (-0.64, -0.182)	<0.001

Supplementary Figures

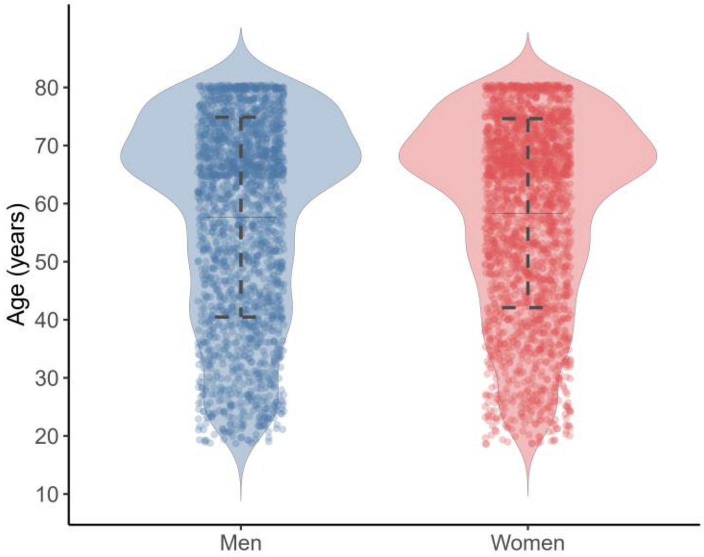

Figure S1. Age distribution of the study population by sex

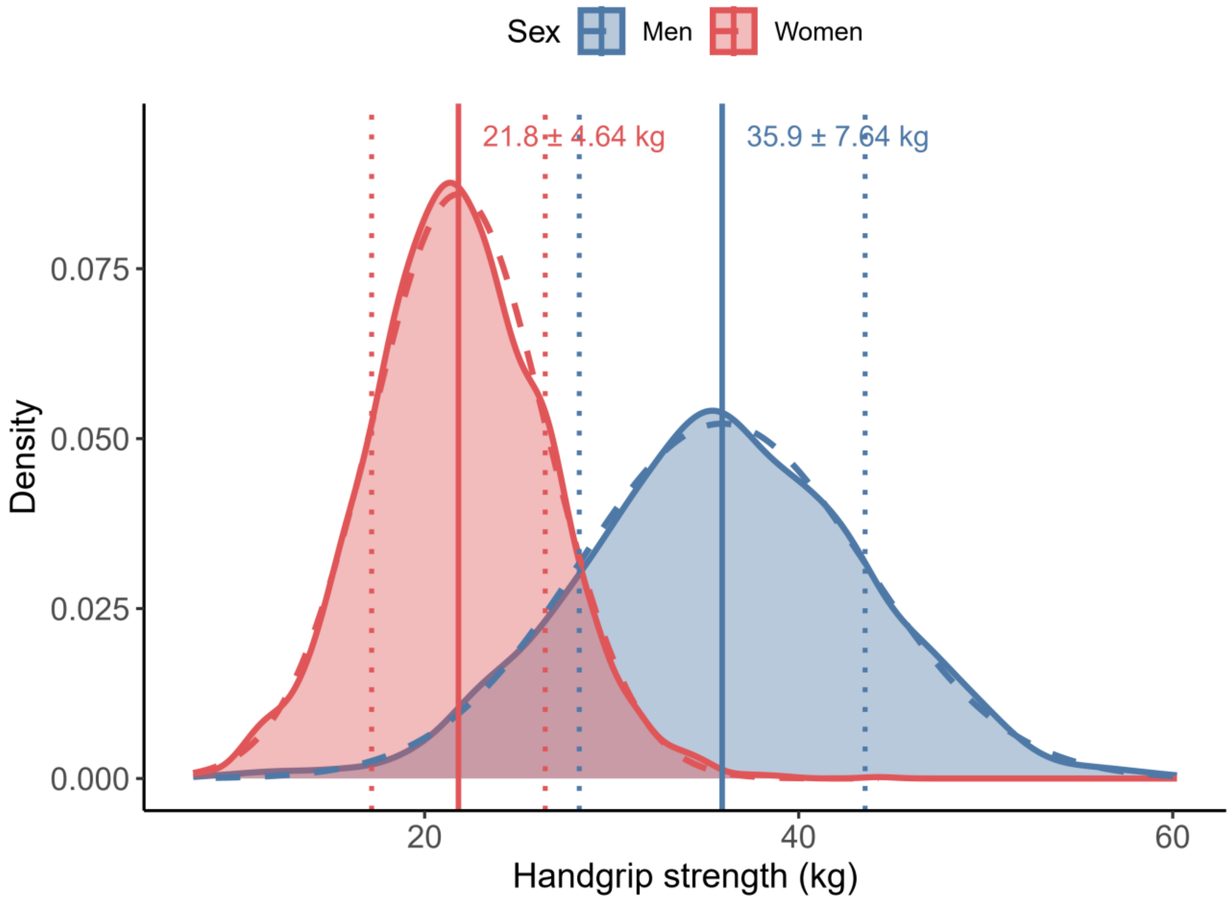

Figure S2. Distribution of handgrip strength by sex

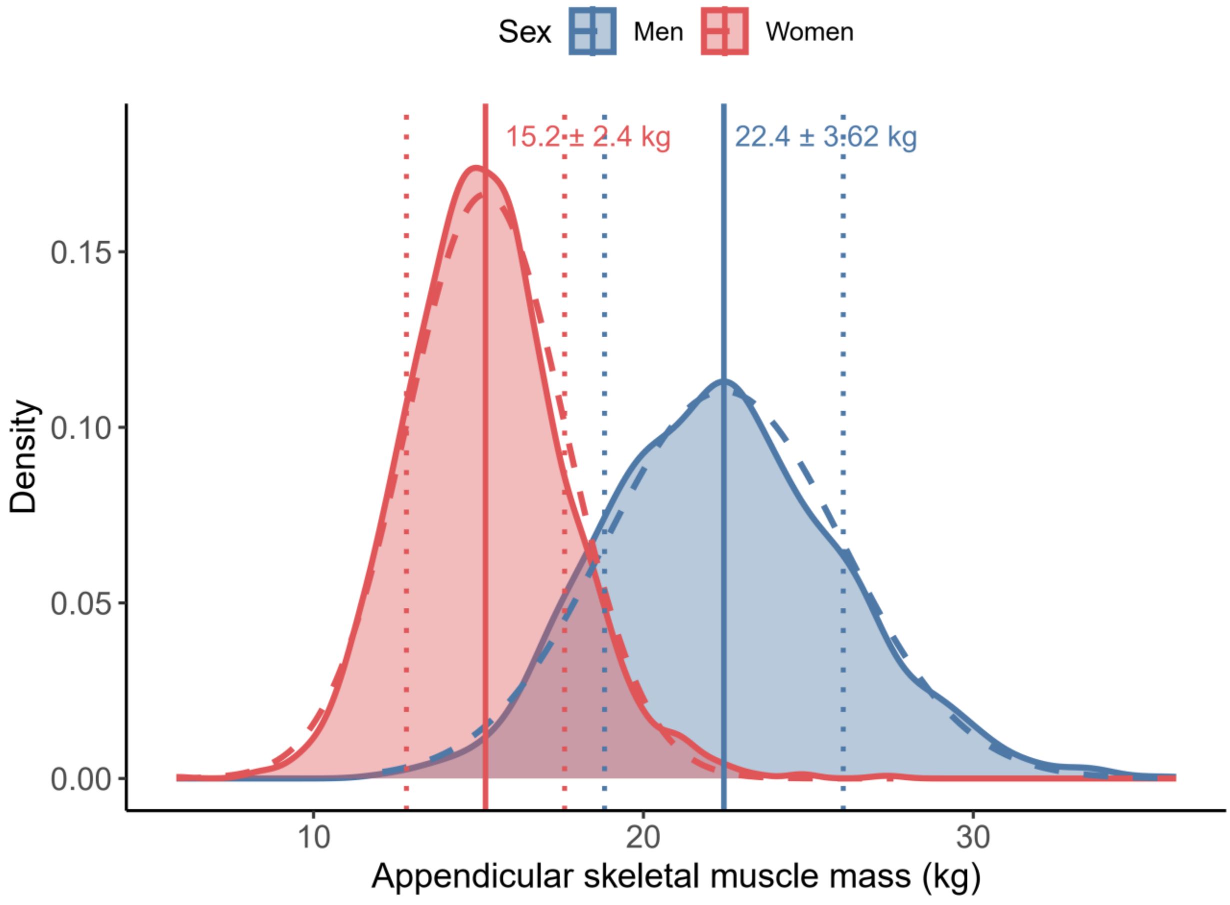

Figure S3. Distribution of appendicular skeletal muscle mass by sex

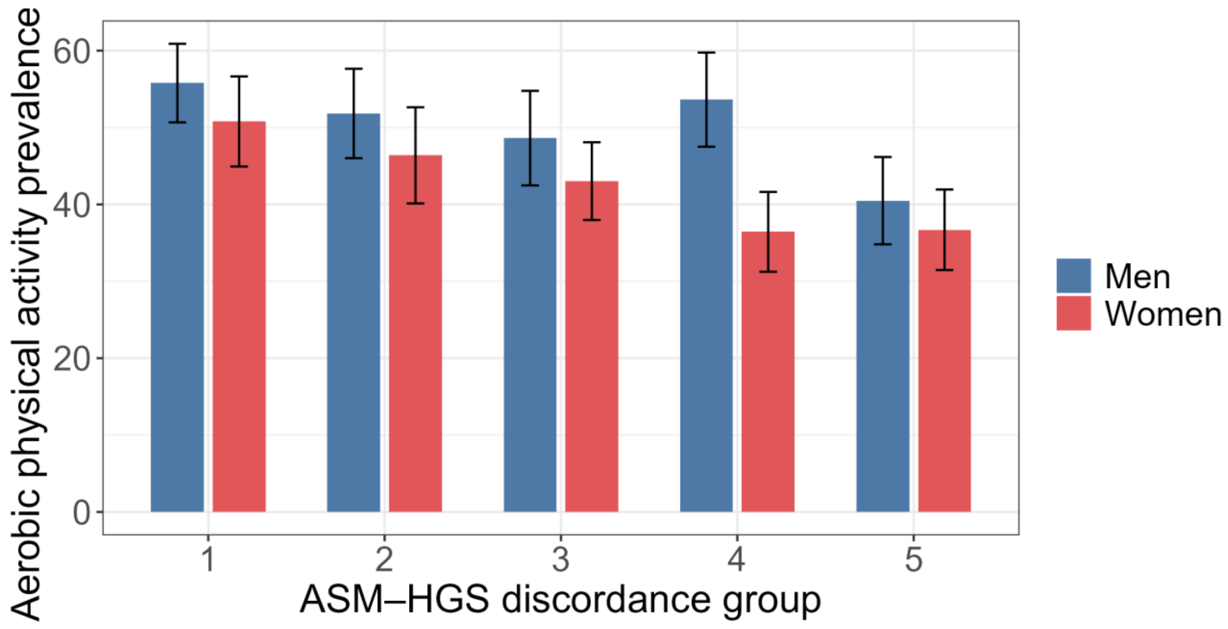

Figure S4. Survey-weighted prevalence of aerobic physical activity according to ASM–HGS discordance group, stratified by sex

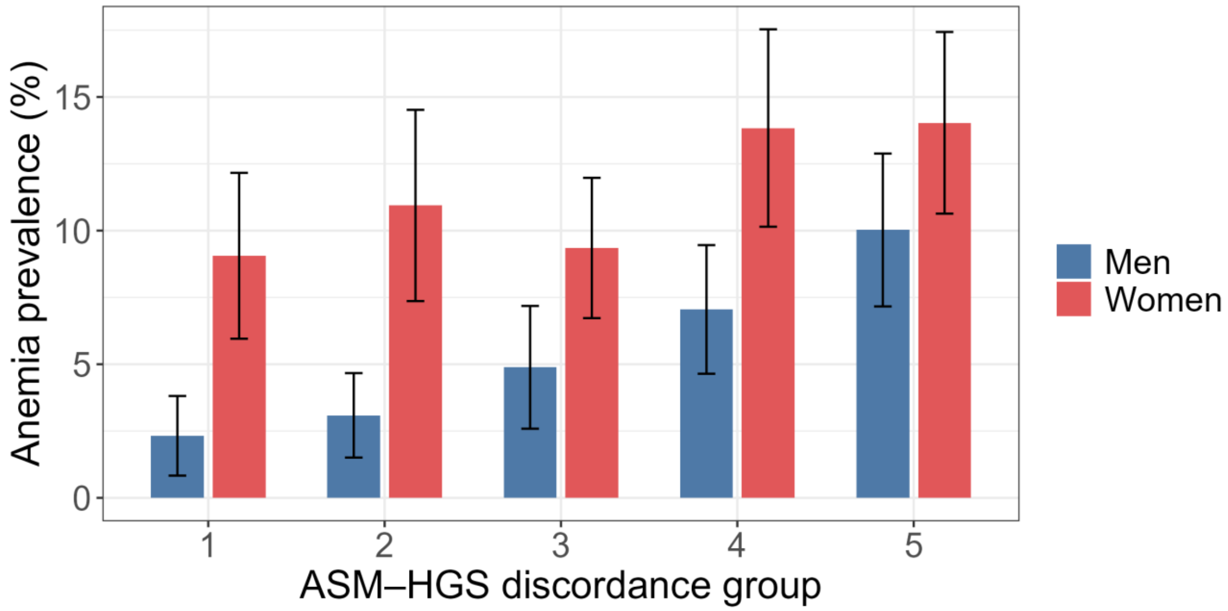

Figure S5. Survey-weighted prevalence of anemia according to ASM–HGS discordance group, stratified by sex

a

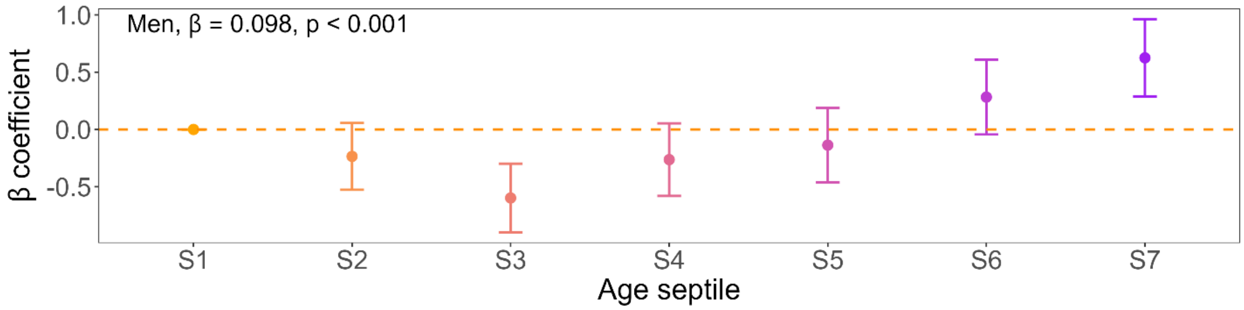

b

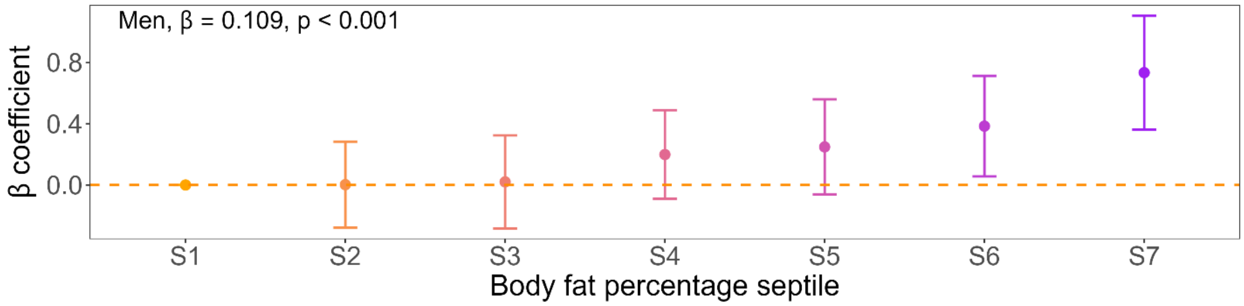

c

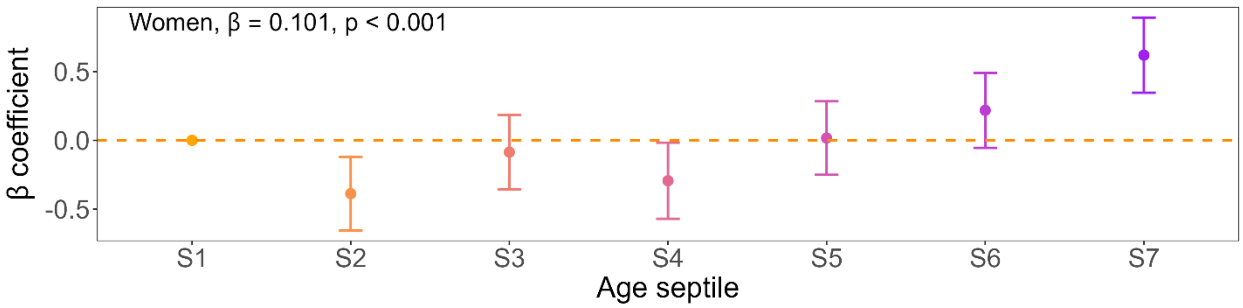

d

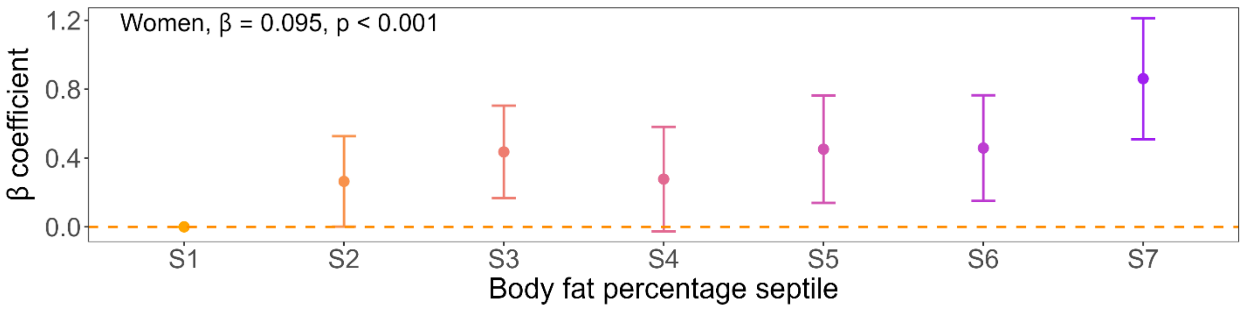

Figure S6. Multivariable-adjusted associations of age, body fat percentage, hemoglobin, and total cholesterol with ASM-HGS discordance (septile)

Panels show the associations between (a) age and ASM–HGS discordance in men, (b) body fat percentage and ASM–HGS discordance in men, (c) age and ASM–HGS discordance in women, (d) body fat percentage and ASM–HGS discordance in women, β-coefficients (points) and 95% con-fidence intervals (error bars) are presented for each septile (S1–S7) of the exposure variables, estimated using multivariable linear regression models adjusted for all covariates that were sta-tistically significant in univariable analyses.

a

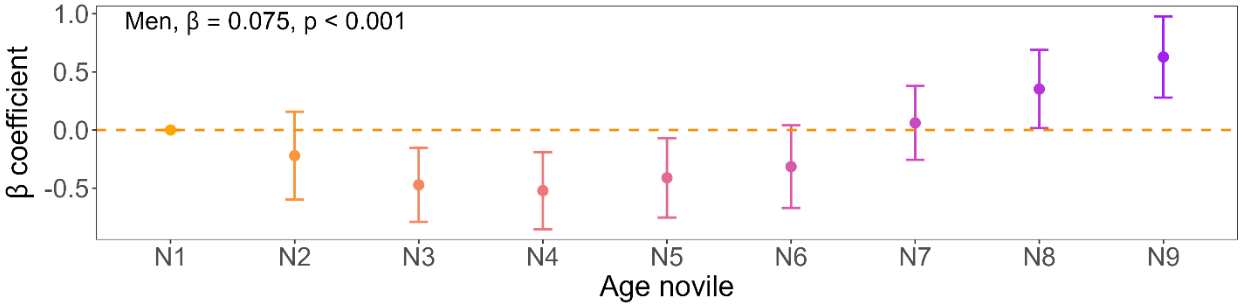

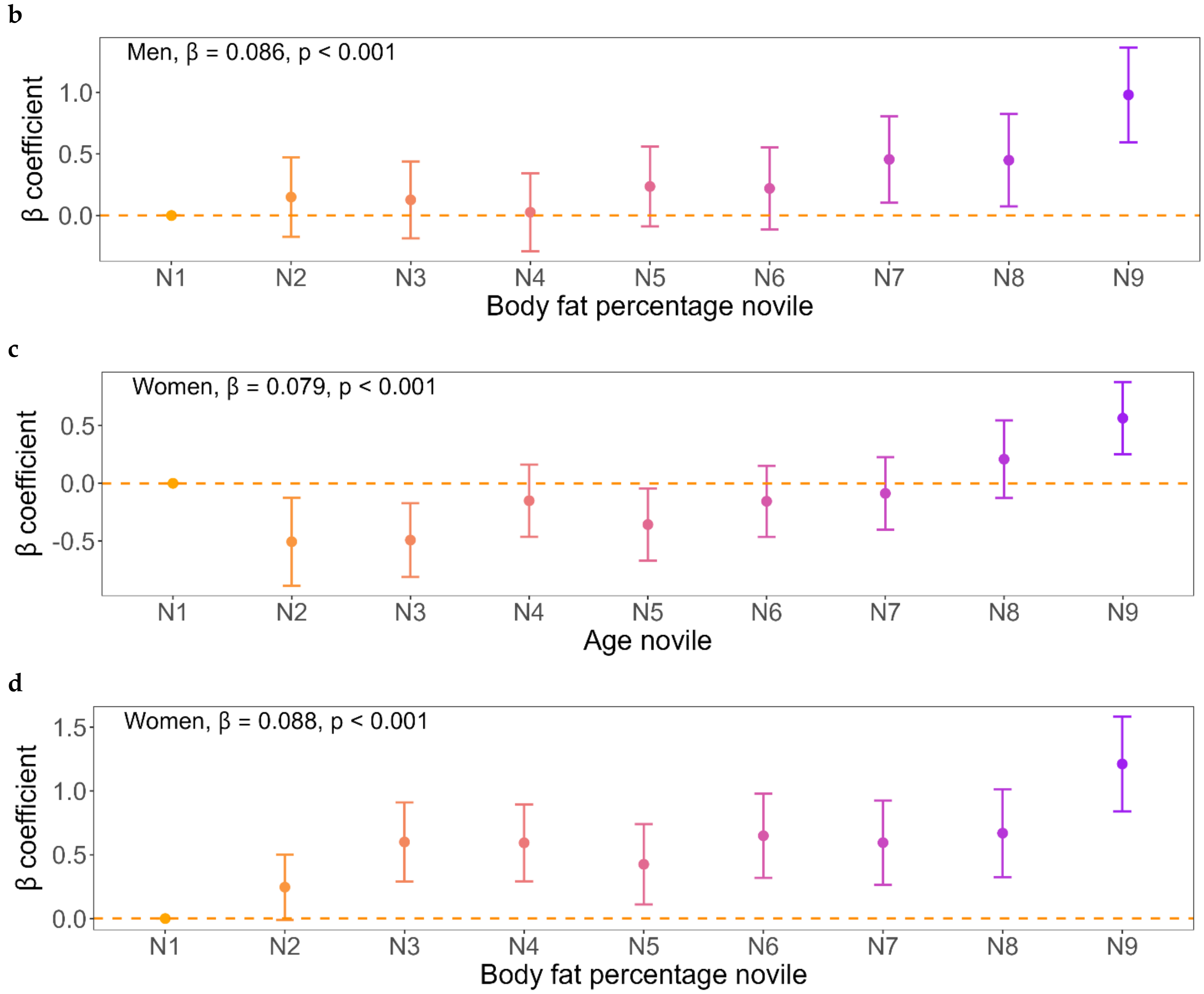

Figure S7. Multivariable-adjusted associations of age, body fat percentage, hemoglobin, and total cholesterol with ASM-HGS discordance (novile)

Panels show the associations between (a) age and ASM–HGS discordance in men, (b) body fat percentage and ASM–HGS discordance in men, (c) age and ASM–HGS discordance in women, (d) body fat percentage and ASM–HGS discordance in women, β-coefficients (points) and 95% con-fidence intervals (error bars) are presented for each novile (N1–N9) of the exposure variables, estimated using multivariable linear regression models adjusted for all covariates that were sta-tistically significant in univariable analyses.
